# The protective effects of antigen-specific IgY on pyocyanin-treated human lymphoma Raji cells

**DOI:** 10.12688/f1000research.19327.2

**Published:** 2019-12-17

**Authors:** Heni Susilowati, Sidna Artanto, Heribertus Dedy Kusuma Yulianto, Wihaskoro Sosroseno, Suryani Hutomo

**Affiliations:** 1 Department of Oral Biology, Faculty of Dentistry, Universitas Gadjah Mada, Sleman, Yogyakarta, 55281, Indonesia; 2 Department of Microbiology, Faculty of Veteriner, Universitas Gadjah Mada, Sleman, Yogyakarta, 55281, Indonesia; 3 Department of Dental Biomedical Science, Faculty of Dentistry, Universitas Gadjah Mada, Sleman, Yogyakarta, 55281, Indonesia; 4 Faculty of Dentistry, AIMST University, Bedong, Kedah, 08100, Malaysia; 5 Department of Microbiology, Faculty of Medicine, Dutawacana Christian University, Yogyakarta, 55225, Indonesia

**Keywords:** Pseudomonas aeruginosa, pyocyanin, IgY, protective effect

## Abstract

**Background:** Pyocyanin (PCN), a highly pathogenic pigment produced by *Pseudomonas aeruginosa*, induces caspase 3-dependent human B cell (Raji cells) death. The aim of the present study, therefore, was to assess whether antigen-specific IgY antibodies may be protective on PCN-induced Raji cell death.

**Methods:** Chickens were subcutaneously immunized with Freund's complete adjuvant containing PCN, and then given two boosted immunizations.  Anti-PCN IgY antibodies were purified from egg yolk and detected using an agar gel precipitation test (AGPT) and ELISA. Protective effects of antigen-specific IgY on Raji cells were tested using a cell viability assay.

**Results:** AGPT results showed the formation of strong immune complex precipitates, whilst ELISA further confirmed the presence of IgY antibodies specific to PCN at significant concentration. Further experiments showed that anti-PCN IgY antibodies significantly increased PCN-treated Raji cell viability in a dose-dependent fashion (p<0.05).

**Conclusions:** The results of the present study suggest that anti-PCN IgY antibodies may be protective on PCN-induced Raji cell death.

## Introduction

*Pseudomonas aeruginosa,* an opportunistic Gram-negative bacterium, is found in the environment with a broad spectrum of habitats and is responsible for severe nosocomial infections in the urinary tract ^1^, the respiratory tract ^2^, the vascular system ^3^ and the central nervous system ^4^. It is known for one of the most common pathogens infecting patients with cystic fibrosis, leading to increase its morbidity and mortality due to the resisting abilities of this pathogen to against antibiotic treatments ^5, 6^. The presence of *P. aeruginosa* in dental pulp and periapical lesions may cause failure of endodontic treatments ^7, 8^. In the initial stage of infection, *P. aeruginosa* releases various virulent mediators, such as elastases, proteases, exotoxin A, and pyocyanin (PCN), after which chronic infection and persistent bacterial colonization at the *P. aeruginosa*-infected sites would be established ^9^. PCN, a blue redox-active secondary metabolite and a member of tricyclic phenazine family, is known to function as a gene controller during the stationary growth phase ^10^, an antibiotic ^11^, an electron transfer facilitator ^12^, and a potent mammary cell-damaging virulence factor ^13^. Reports indicate that PCN inhibits B cell, T cell and macrophage functions ^14, 15^ and induces neutrophil apoptosis ^16^, suggesting that PCN suppresses both innate and antigen-specific adaptive immune response.

The existence of multidrug-resistant (MDR) *P. aeruginosa* leads to the development of alternative treatment strategies to eradicate an established chronic *P. aeruginosa* infection. Of these treatments, both active and passive immunotherapies have been reported. Active immunization with *P. aeruginosa*-derived flagella in cystic fibrosis patients resulted in increased serum antigen-specific IgG antibodies and reduced number of *P. aeruginosa* strains, suggesting the reduction of *P. aeruginosa* infection risk in cystic fibrosis patients by active vaccination ^17^. Passive immunization with egg yolk immunoglobulin (IgY) specific to *P. aeruginosa* in patients with cystic fibrosis prevented bacterial colonization and infection, perhaps by acting as an opsonin, which in turn enhanced neutrophil phagocytosis to this pathogen ^18– 20^. A recent study showed that PCN induces caspase 3-dependent human B cell (Raji Cells) death ^21^. The aim of the present study, therefore, was to determine whether antigen-specific IgY antibodies may prevent PCN-induced Raji cell death.

## Methods

### IgY preparation and purification

PCN (Sigma-Aldrich, St. Louis, MO, USA) was dissolved in DMSO at a concentration of 1 mg/ml. Five Leghorn chickens aged 3 months were subcutaneously immunized with 500 μl of Freund's complete adjuvant (Sigma-Aldrich) containing 100 μg of PCN in the back of the neck. Two weeks later, a booster was given by injecting 500 μl incomplete Freund adjuvant containing 40 μg PCN as above and the same immunization regime was repeated two weeks later. Eggs were collected one week after the last immunization and IgY was isolated by using Pierce® Chicken IgY Purification Kit (Thermo Fisher Scientific Pierche Biotechnology, Rockford, USA) according to the manufacturer. The presence of anti-PCN IgY antibodies was detected using the agarose gel precipitation test (AGPT) as previously reported ^22^ and its concentration was assessed using the Chicken IgY ELISA Kit (Elabscience Biotechnology Co., Ltd, USA). The AGPT test was performed three times, each of 4 isolates from the first and second IgY purification results. The ELISA was then performed on two IgY batches.

### Cell cultures

Raji cells, a human B cell line, obtained from central university laboratory LPPT, Universitas Gadjah Mada, Yogyakarta, Indonesia, were cultured in RPMI 1640 medium supplemented with 10% fetal bovine serum, 100 UI/ml of penicillin-streptomycin, and 250 μl/ml of amphotericin B and then incubated in 5% CO _2_ humidity. All materials for culture medium were purchased from Sigma-Aldrich. The cells were cultured in 96-well plates and five replicates were carried out for assays.

### Cell viability

PCN (Sigma-Aldrich) was initially dissolved in DMSO (Sigma-Aldrich) at a concentration of 1 mg/ml and then diluted in RPMI to a final concentration of 1 μg/ml, 10 μg/ml, 25 μg/ml, and 50 μg/ml. Raji cells at 2 × 10 ^4^ cells incubated without the presence of PCN were used as a negative control. After exposure to various concentration of PCN then the cultures were incubated at 37°C for 24 hours. In the next experiments, the cells, at a concentration of 2 × 10 ^4^ cells/well, were treated with 50 μg/ml PCN with or without the presence of various concentration (6.71 μg/ml, 13.42 μg/ml, 28.19 μg/ml, 55.49 μg/ml, 111.87 μg/ml, and 223.75 μg/ml) of anti-PCN IgY were cultured in 96-well plates and incubated for 16 hours. Cell survivability was assessed by MTT assay as described previously ^21^. Experiments were carried out three times with 8 replicates in each group.

In order to assess cell viability, 5 × 10 ^4^ cells/well were cultured on sterile coverslips in 24-well plates for 24 hours and then treated with PCN in the presence or absence of anti-PCN IgY (55.49 µg/ml) for 16 hours. Subsequently, the cells were stained with acrydine orange/ethidium bromide and viewed under Digital Carl Zeiss-Axioscope 40 (Carl Zeiss Vision, Oberkochen, Germany) by which viable and death cells appeared as green and orange/red, respectively.

### Statistical analysis

The results of PCN cytotoxicity assay on Raji cells were analyzed by using one way analysis of variance followed by LSD test. Data obtained from the experiments on the effects of anti-PCN IgY on PCN-treated Raji cells was analysed by using one-way ANOVA followed by Tukey’s Test. Statistical analysis was calculated by using IBM SPSS Statistics Version 22 (SPSS Inc., IBM Corp., Chicago, IL).

## Results

**Figure 1.**
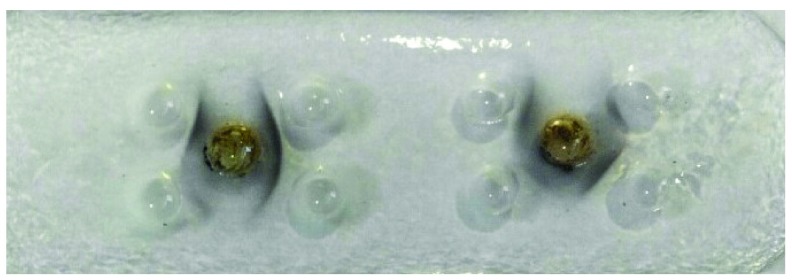
Agarose gel precipitation test of pyocyanin (PCN)-IgY antibody complex batch I and II. The presence of PCN-IgY antibody was detected through the presence of precipitates formed on agarose gel.

Following isolation and purification of IgY from the egg immunized chickens, PCN-IgY complexes were detected by using AGPT. As seen in Figure 1, clear lines of precipitates from two IgY batches in the agarose matrix indicated the presence of PCN-specific antibodies. A further assessment using ELISA demonstrated that the first batch gives high amount of specific IgY antibodies (8.95 μg/μl) than that one of the second (3.02 µg/µl) which were then used for the rest of experiments.

**Figure 2.**
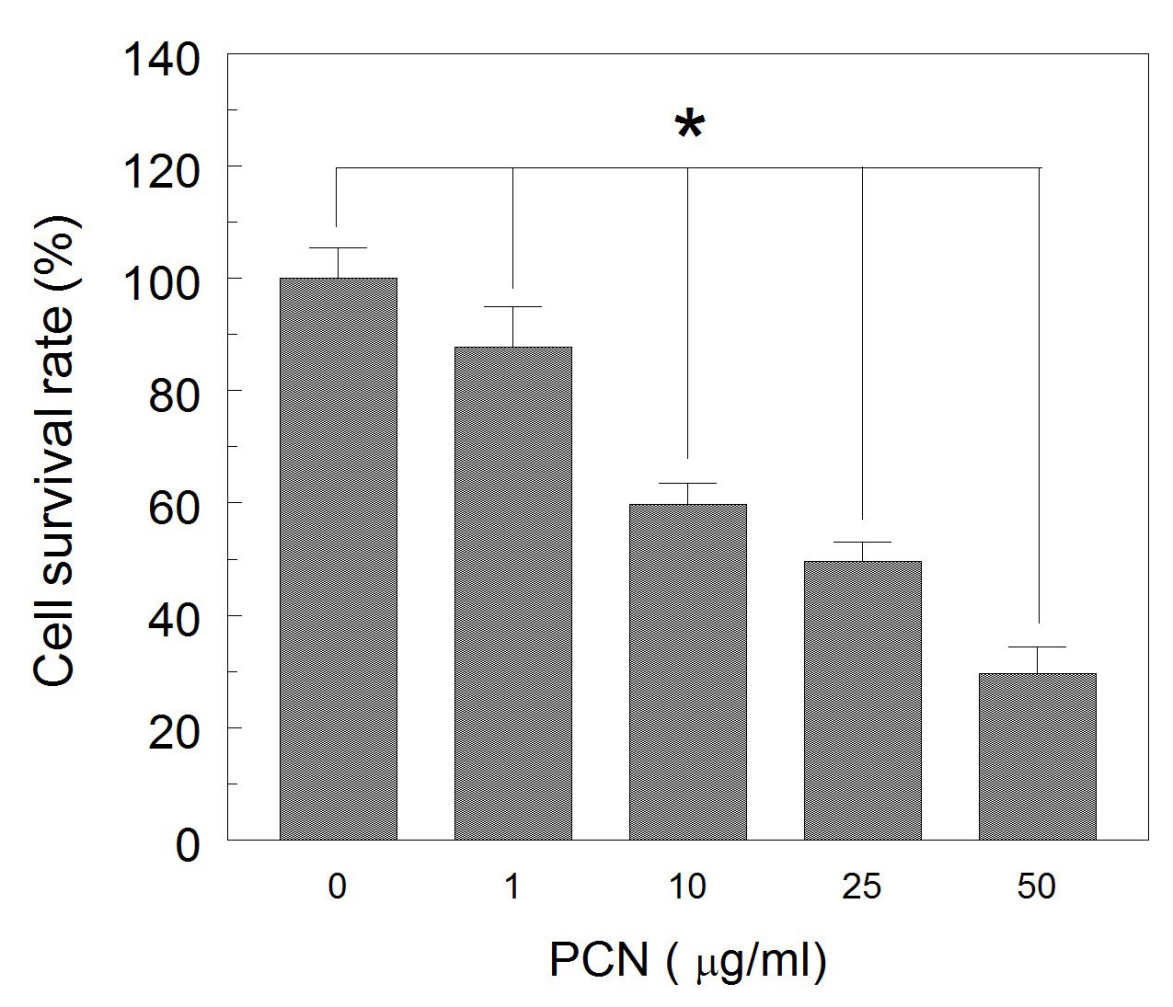
The effects of pyocyanin (PCN) on Raji cell survivability. After incubation with various concentration of PCN, Raji cell viabilities were assessed by MTT assay. PCN-treated Raji cell survivability was calculated against the control cells. *p<0.05.

The results of this study showed that PCN at 1 mg/ml was toxic to the Raji cells. This cytotoxic effect of PCN on the cells was steadily increased in a dose dependent fashion (p<0.05) ( Figure 2).

**Figure 3.**
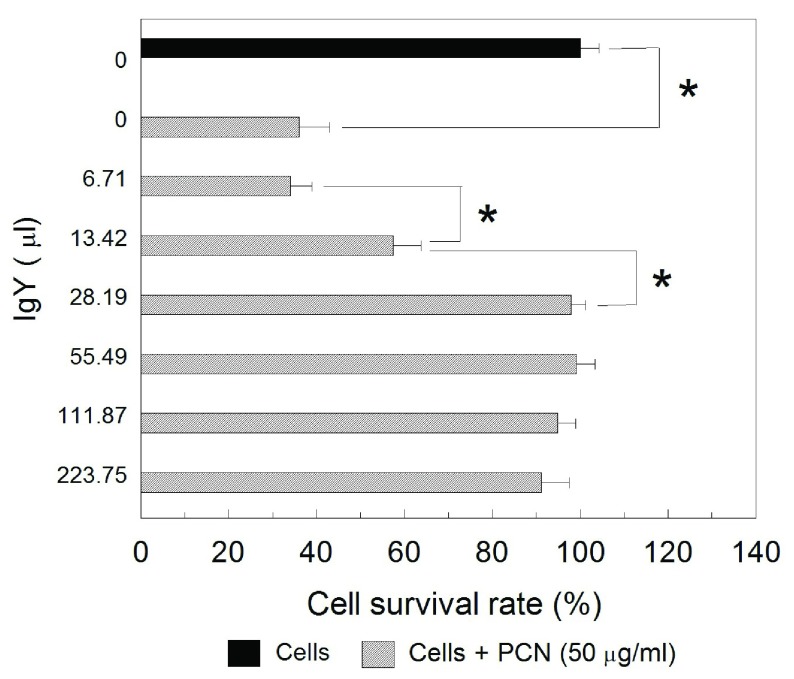
The effects of IgY specific antibodies on pyocyanin (PCN)-treated Raji cell survivability. PCN was incubated with various concentration of IgY antibodies. Raji cells were then incubated with the PCN-IgY mixtures. Viable cells were assessed by MTT and their percentage was calculated as in Figure 2. *p<0.05.

**Figure 4.**
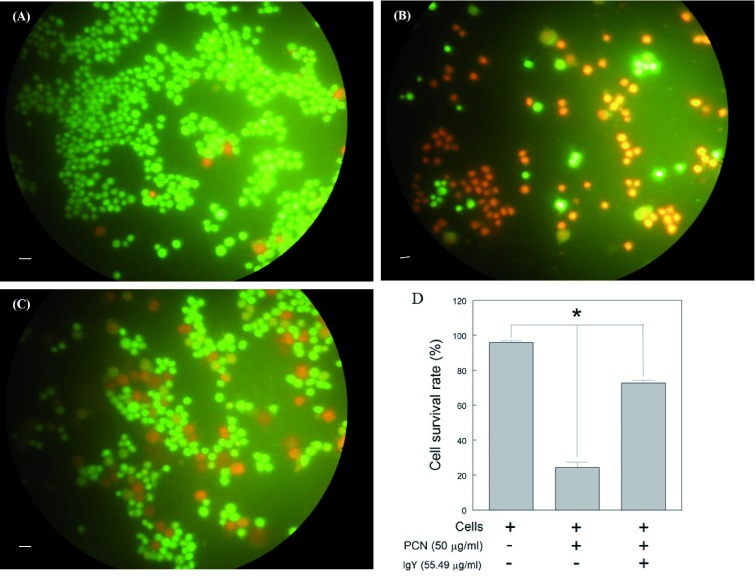
Microscopic features of Raji cells treated with pyocyanin (PCN) or PCN-IgY antibody complexes. Raji cells were incubated without PCN ( **A**) and with PCN ( **B**) or the mixture of PCN-IgY antibodies ( **C**) and then stained with acridine orange/ethidium bromide. Green or orange fluorescence stained cells are viable and dead cells, respectively. The cell survivability was increased on the cells treated with IgY antibody prior to PCN exposure (D).

Further experimentation demonstrated that anti-PCN IgY at concentrations of 28.19 μg/mL or higher was able to suppress the cytotoxic effect of PCN on Raji cells as compared with the negative control (p<0.05) ( Figure 3). No significant differences between the cells treated with PCN and specific anti-PCN IgY antibodies at the concentration above 55 μg/ml were observed, however (p>0.05) ( Figure 3). Microscopically, the number of viable cells treated with PCN-IgY complexes was much higher than those treated with PCN only ( Figure 4). The results showed that anti-PCN IgY did increase the survivability of PCN-exposed cells from 24.3% up to 72.6% at concentration at 55.49 μg/ml ( Figure 4D). Statistically, significant differences existed between cell only and PCN-treated or PCN-IgY-treated cells. The survivability of cells treated by PCN was significantly lower than that treated with IgY prior to PCN stimulation (p<0.05). Raw cell viability counts, along with other raw results and images, are available as *Underlying data* ^23^.

## Discussion

The results of the present study showed that PCN does induce cell death in Raji cells as also seen in our previous report Susilowati. Similarly, other also demonstrated that PCN plays an important role in *P. aeruginosa* pulmonary infection through the induction of neutrophil cell death, which involves the release of reactive oxygen species and the activation of mitochondrial acid sphingomyelinase ^16^. Therefore, efforts to inhibit excessive host cell damage induced by PCN are imminent.

Further results of the present study demonstrated that anti-PCN IgY antibodies specific to PCN significantly reduce the ability of this pathogen to induce Raji cell death in a dose-dependent fashion. Whilst no previous studies showing prevention of PCN-induced cell death by antigen-specific IgY have yet been reported to our knowledge, the present results are supported by the fact that antigen-specific IgY antibodies did prevent *P. aeruginosa* infection in humans by both active and passive immunization ^17– 19^, suggesting that *P. aeruginosa*-specific IgY antibodies may inhibit cellular inflammatory responses induced by this pathogen. Pyocyanin is secreted by *P. aeruginosa* when the nutrition for the bacterium is limited. As important virulent factor, PCN plays a key role in bacterial infection because its ability to cross cell membrane which then causing disturbance in cellular electron transport and innate immune response ^24^. Since PCN functions to kill host cells ^25^ it is possible to inhibit cellular and tissue damage by blocking PCN using the anti PCN-antibody IgY. Antigen-specific IgY antibodies also stimulated *P. aeruginosa* aggregation and increased human neutrophil phagocytic activities ^20^. The exact mechanism by which antigen-specific IgY antibodies inhibited PCN-induced Raji cell death seen in the present study remains unclear, however. Zhao *et al.* demonstrated the activation of caspase-3 in hepatoma HepG2 cell death mechanism induced by PCN ^26^. Other has shown that PCN stimulates NK cell death via increased intracellular calcium levels and mitochondrial disruption ^27^, suggesting that cell death induced by PCN may utilize different apoptotic signal pathways, perhaps, in a cell type-dependent fashion. Our previous study indicated that PCN induced Raji cell death via a caspase 3-activation pathway ^21^. It seems plausible, therefore, that PCN-IgY antibody complexes may fail to activate Raji cell-derived caspase 3 and hence, inhibit cell death. However, more studies are required to delineate this speculation.

 The extrapolation of the results of the present study in the therapy of *P. aeruginosa* infection remains to be further investigated. *P. aeruginosa* with its multiple mechanisms for adaptation and survival is well known as one of the main pathogens that lead to increase nosocomial infections ^1– 4^ and morbidity and mortality in cystic fibrosis patients ^5^. The difficulty to eradicate *P. aeruginosa* infection has been even more complicated by the presence of MDR *P. aeruginosa*, and hence alternative supplemental treatment approaches have been put forward based upon its bacterial virulent factors, such as exotoxin, lipopolysaccharide, and flagellin ^28^. Immunotherapies using IgY specific to *P. aeruginosa* to delay initial infection and reduce both frequency of infection and development of chronic infection seem to be promising. Both passive and active immunization with antigen-specific IgY antibodies in humans resulted in inhibition of *P. aeruginosa* infection. For example, oral immunization with specific IgY antibodies in patients with cystic fibrosis led to the inhibition of *P. aeruginosa* colonization ^18, 19^, suggesting the usefulness of antigen-specific IgY antibodies an immunotherapy to prevent *P. aeruginosa* infection in patients with cystic fibrosis. Therefore, PCN-specific IgY antibodies used as an immunotherapy alongside with the common antibiotic treatments for *P. aeruginosa* infection are highly possible.

 In conclusion, the present study showed that eggs from PCN-immunized chickens contain substantial amount of IgY antibodies that recognize PCN. Furthermore, IgY antibodies derived from PCN-immunized chicken were able to inhibit PCN-induced Raji cell death, suggesting that PCN-specific IgY antibodies may be protective against PCN-induced Raji cell death. Future studies need to clarify the mechanisms involved in inhibiting the death of Raji cells induced by PCN. Furthermore, it is also necessary to study the effect of IgY antibodies derived from PCN-immunized chicken on the in vivo model of *P. aeruginosa* infection..

## Data availability

Figshare: Cytotoxicity of PCN.xlsx. https://doi.org/10.6084/m9.figshare.8115701.v6 ^23^.

This project contains the following underlying data:

- Cytotoxicity of PCN.xlsx (raw cell viability data following treatment with pyocyanin)
- The effect of IgY on cell viability.xlsx (raw cell viability data following treatment with pyocyanin and IgY)
- Fig 4A untreated cells.JPG (raw image used for Figure 4A)
- Fig 4B PCN-treated cells.JPG (raw image used for Figure 4B)
- Fig 4C PCN IgY-treated cells.JPG (raw image used for Figure 4C)
- Fig 4D Cell survivability (raw image used for Figure 4D)
- Acridine orange-ethidium bromide results (raw cell viability data)
- IMG-AGPT.jpg (raw image of agar gel precipitation test)
- Elisa results Sept 1.xls (raw ELISA data)

Data are available under the terms of the Creative Commons Attribution 4.0 International license (CC-BY 4.0).
