# Supplementary material for: The protective effects of antigen-specific IgY on pyocyanin-treated human lymphoma Raji cells
Source: F1000Res. 2019 Dec 17;8:1008. Originally published 2019 Jul 4. [Version 2] doi: 10.12688/f1000research.19327.2 (PMC6971839; doi:10.12688/f1000research.19327.2)
Supplement: Peer review history

# Peer review: Reviewer response for version 2

**DOI:** 10.5256/f1000research.23846.r57905

**Published:** 2019-12-20

**Authors:** Boy M. Bachtiar

**Affiliations:** 1 Department of Oral Biology and Oral Science Research Center, Faculty of Dentistry, Universitas Indonesia, Jakarta, Indonesia

I confirm that I have read this submission and believe that I have an appropriate level of expertise to confirm that it is of an acceptable scientific standard.

# Response

**Published:** 2020-01-13

**Authors:** Heni Susilowati

**Affiliations:** Universitas Gadjah Mada, Indonesia

Dear Dr. Boy M Bachtiar,

We express our high appreciation for the review, critical analysis, and suggestions given for the improvement of our manuscript. We hope this research report will be useful for the development of science in the health field.

# Peer review: Reviewer response for version 1

**DOI:** 10.5256/f1000research.21186.r52110

**Published:** 2019-08-27

**Authors:** Priya Madhavan

**Affiliations:** 1 School of Medicine, Faculty of Health and Medical Sciences, Taylor’s University, Subang Jaya, Malaysia

Overall, the study design is of an acceptable quality. Immunotherapy using IgY specific to *P. aeruginosa* looks promising. Minor English editing and grammar check are required.

- Magnification/Scale on Figure 4 is missing. Best to include this.
- Why isn't there a positive control? I can only see results with negative control. Please provide a justification for not including a positive control.
- Best to also include other articles from year 2018-2019.

I confirm that I have read this submission and believe that I have an appropriate level of expertise to confirm that it is of an acceptable scientific standard.

# Peer review: Reviewer response for version 1

**DOI:** 10.5256/f1000research.21186.r50799

**Published:** 2019-07-16

**Authors:** Boy M. Bachtiar

**Affiliations:** 1 Department of Oral Biology and Oral Science Research Center, Faculty of Dentistry, Universitas Indonesia, Jakarta, Indonesia

**General comments:**

 In this manuscript, the authors provide data account of an IgY-antibody that was purified from eggs of hens immunized with PCN secreted by *P. aeruginosa*. The specificity of PCN-IgY binding was determined by using AGPT, and it was further examined in vitro experiment to mimic the naturally occurring of bacterium-host cell interaction. In my opinion, it is necessary to explain (in the discussion session), when the toxin (PCN) is secreted and injected into host cells. For example, after cell contact (adherence phase).

**Specific commons:**

**Methods:**

1. In the experiment, PCN was added into the cultured cell in the presence or absence of anti-PCN IgY (acted as control). This means, in the absence of the IgY, the toxin can enter the host cell (might be in adherence phase) due to the presence of a receptor, then induced the intracellular pathway leading to human B cell (Raji Cells) death (21). If this is the case, please explain it in the discussion session.

**Results:**

1.  In order to exclude chicken serum as the other IgY source, please change “the immunized chickens” to “egg of immunized chickens”.
2. In this study, they used a microscope (fluorescence microscope?) to compare the Raji cells number between groups tested, qualitatively, thus the obtained results was not quantitative.  

**Discussion:**

1. ”anti-PCN IgY antibodies specific ………….to this virulence”. I suggest changing this virulence with this pathogen.
2. “the present results are supported by the fact that antigen-specific IgY antibodies did prevent *P. aeruginosa* infection…….., suggesting that *P. aeruginosa*-specific IgY antibodies may inhibit cellular inflammatory responses induced by this pathogen”. Since the IgY used in this study was specifically bind to the secreted PCN, not to bacterium’s whole cell, the rationale behind using this antibody is need to be explored.
3. According to the reference (16), the toxin is not having a role in the bacterial invasion into the host cell, but it involves in the mechanisms by which the bacterium kills the host cell tested (neutrophils).
4. “Furthermore, antigen-specific IgY antibodies were……, suggesting that PCN-specific IgY antibodies may be protective against PCN-induced Raji cell death”. Not clear, what (cell surface) antigen/s that the authors assumed to bind specifically to the IgY, in addition to the secreted toxin (PCN).
5. Based on this study, please suggest, what should be done in future studies.

I confirm that I have read this submission and believe that I have an appropriate level of expertise to confirm that it is of an acceptable scientific standard, however I have significant reservations, as outlined above.
